# The Effect of Aromatherapy Abdominal Massage on Alleviating Menstrual Pain in Nursing Students: A Prospective Randomized Cross-Over Study

**DOI:** 10.1155/2013/742421

**Published:** 2013-04-11

**Authors:** Tyseer M. F. Marzouk, Amina M. R. El-Nemer, Hany N. Baraka

**Affiliations:** ^1^Department of Maternity and Gynecology of Nursing, Faculty of Nursing, Mansoura University, Mansoura 35516, Egypt; ^2^Department of Pharmacognosy and Medicinal Plants, Faculty of Pharmacy, Mansoura University, Mansoura 35516, Egypt

## Abstract

Dysmenorrhea is a common cause of sickness absenteeism from both classes and work. This study investigated the effect of aromatherapy massage on a group of nursing students who are suffering of primary dysmenorrhea. A randomized blind clinical trial of crossover design was used. In the first treatment phase, group 1 (*n* = 48) received aromatherapy abdominal massage once daily for seven days prior to menstruation using the essential oils (cinnamon, clove, rose, and lavender in a base of almond oil). Group 2 (*n* = 47) received the same intervention but with placebo oil (almond oil). In the second treatment phase, the two groups switched to alternate regimen. Level and duration of pain and the amount of menstrual bleeding were evaluated at the baseline and after each treatment phase. During both treatment phases, the level and duration of menstrual pain and the amount of menstrual bleeding were significantly lower in the aromatherapy group than in the placebo group. These results suggests that aromatherapy is effective in alleviating menstrual pain, its duration and excessive menstrual bleeding. Aromatherapy can be provided as a nonpharmacological pain relief measure and as a part of nursing care given to girls suffering of dysmenorrhea, or excessive menstrual bleeding.

## 1. Introduction

Dysmenorrhea is one of the most common gynecologic disorders affecting more than half of menstruating women [1]. It is defined as a pelvic pain directly related to menstruation that interferes with daily activities [2]. Dysmenorrhea is classified into two categories: primary dysmenorrhea which is a cramping pain in the lower abdomen occurring just before or during menstruation, when pelvic examination and ovulatory function are normal, and secondary dysmenorrhea, which refers to painful menses when there is an identifiable gynecological pathology such as endometriosis  [3]. Prevalence of primary dysmenorrhea is reported in many studies to vary between 50% and 90% [4]. In an epidemiological study that entailed 664 secondary school students from urban and rural areas in Mansoura, Egypt. It was found that about 75% of the students have dysmenorrhea, rated mild in 55.3%, moderate in 30%, and severe in 14.7% [5]. Primary dysmenorrhea starts when adolescents attain their ovulatory cycles, generally within three years of menarche [3]. The etiology of primary dysmenorrhea is not precisely understood; the pain is believed as the result of excessive prostaglandin release, particularly PGF2 *α* [6]. PGF2 *α* causes vasoconstriction of uterine blood vessels (uterine ischemia) and increases uterine smooth muscle contraction, and it is the contraction of the ischemic uterus that is likely the cause of dysmenorrhea [7]. Within the Egyptian culture, dysmenorrhea is not acknowledged as a health problem which needs medical or nursing intervention [8]. Egyptian young girls are not preferring to use medication for dysmenorrhea as they believe that it may affect fertility or causing some types of dependence [9]. Consequently, dysmenorrhea is not managed effectively despite its high occurrence [10]. Researchers have investigated different alternative therapies that are effective and safe and can be prescribed by nurses. A variety of alternative methods have been used for the treatment of dysmenorrhea such as acupuncture, Transcutaneous Electronic Nerve Stimulation (TENS), biofeedback, herbal therapy, and complementary medicine [11–13]. Aromatherapy is the most widely used complementary therapy in nursing practice and uses essential oils from fragrant plants to relieve health problems and improve quality of life in general [14]. Essential oils can be used in different ways, including massage, bathing, and inhalation. When essential oils are inhaled, olfactory receptor cells are stimulated and the impulses are transmitted to the emotional centre of the brain, or “limbic system.” The properties of the oils, the fragrance, and its effects, determine stimulation of these systems. When used in massage, essential oils are not only inhaled but also absorbed through the skin as well. They penetrate the tissues and find their way into the bloodstream where they are transported to the organs and systems of the body [15–17]. Different oils are thought to act on the body in different ways, having a relaxing, energizing, calming, or uplifting effect. Aromatherapy is inexpensive and safe to relief dysmenorrhea. Also, it improves blood circulation and reduces the spasms compared with conventional therapy [18, 19]. However, dysmenorrhea is common; its management has been inadequately addressed. The purpose of this study was to investigate the effect of aromatherapy abdominal massage on alleviating menstrual pain and reducing its duration and excessive menstrual bleeding. 

## 2. Subjects and Methods

### 2.1. Study Design

This is a randomized blind clinical trial of crossover design. Students were assessed three times during the course of the study: at a baseline (at the first menstrual period after enrollment prior to starting intervention) and during the menstrual periods after each treatment phase. After baseline assessment, the students were then assigned randomly into two groups: 48 students in group 1 and 47 students in group 2. After randomization, the first treatment phase was started (at the second menstruation after enrollment). During this phase, students in group 1 (aromatherapy group) received aromatherapy massage using essential oils while students in group 2 (placebo group) received massage with sweet almond oil. In the second treatment phase (at the third menstruation after enrollment,) the intervention has been switched among the two groups. 

### 2.2. Participants

The present study was conducted in the Faculty of Nursing at Mansoura University in Egypt, in the period from October 2010 to January 2011. One hundred eligible nursing students who were suffering of primary dysmenorrhea with a pain level ≥6 on a visual analogue scale were recruited to participate in the study. Students were eligible to participate in the study, if they were single, aged 17–20 year-old at the time of the study, with regular cycles 21 to 35 days lasting three to seven days, and had no systemic or gynecologic disease. Excluded from the study students who were married, on hormonal therapy during the last 6 months, receiving analgesics during the study period, known or suspected secondary dysmenorrhea (major abdominal or pelvic surgery, endometriosis, pelvic inflammatory disease (PID), ovarian cysts, pathological vaginal secretion, chronic abdominal pain, inflammatory bowel disease, and irritable bowel syndrome). Five students were dropped, three of them were excluded from the study as they were taking analgesics during their period and two were unable to follow up with the study regimen. The ninety-five students were randomly allocated into two groups by means of block randomization (block size of 4) using the concealed envelope method. The researcher who is responsible for data collection was blinded to the group's allocation and treatment oils.

### 2.3. Intervention

The study was approved by the Faculty of Nursing Research Ethical Committee at Mansoura University. All students signed a written consent followed by a baseline interview. During the interview, the collected data included student's age, age at menarche, length of menstrual cycle, duration of menstrual flow, level and duration of pain, and amount of menstrual bleeding by counting the number of saturated pads.

At first-phase treatment, aromatherapy group received 10 minutes abdominal massage by the researcher in student's clinic at the Faculty of Nursing, once daily for seven days prior to menstruation using essential oils: cinnamon, clove, rose, and lavender oils in a 1.5 : 1.5 : 1 : 1 ratio, respectively, diluted in sweet almond oil with a final concentration of 5%. 

The placebo group received the same intervention of aromatherapy group with sweet almond oil only. Both essential oils and sweet almond oil were packed in similar bottles with the same color, shape, and size by the pharmacognosist researcher, who was the only one who knows which student belongs to which group according to the number given on each bottle. In the second phase, the intervention has been switched among the two groups.

### 2.4. Outcome Measures

The primary outcome measure was the pain level experienced by the students; it was assessed by visual analogue scale (VAS-pain). It is a simple assessment tool consisting of a 10 cm line with 0 on one end representing no pain, and 10 on the other end representing the worst pain. Secondary outcome measures included the duration of pain (hours) and the amount of menstrual bleeding (number of saturated pads). Outcome measures were evaluated on the first, second, and third days of menstruation for the three assessment points during the course of the study.

### 2.5. Statistical Analysis

The data were analyzed using SPSS for windows version 17.0 (SPSS, Chicago, IL, USA). Continuous data obtained at baseline and after each treatment phase were expressed as mean ± SD and were compared between each two groups using independent Student *t*-test. Categorical data was expressed in number and percent and compared using the *χ* ^2^ test. The 95% confidence intervals (CI) for the difference in means were calculated. Statistical significance was set at *P* < 0.05.

## 3. Results

### 3.1. Students Baseline Data at the Time of Enrollment

Table 1 shows no significant differences among the two groups regarding the baseline data. 

### 3.2. Changes of Pain Level and Duration in the First Treatment Phase

Despite the fact that there were no significant differences among the two groups at baseline evaluation, the first phase of the treatment yielded a significantly lower VAS-pain and pain duration in the first group as compared to the second group (Table 2). The VAS-pain in the first day was 5.8 ± 2.1 in the first group compared to 6.8 ± 1.7 in the second group. This difference was significant (95% CI; −1.7921, −0.2213, *P* = 0.013). In the second day, the VAS-pain was also significantly lower in the first group (4.3 ± 2) than in the second group (5.4 ± 1.9) (95% CI; −1.7359, −0.1502, *P* = 0.010). In the third day, the first group continued to have significantly lower average VAS-pain when compared to the second group (2.7 ± 2 versus 3.8 ± 1.9, resp.) (95% CI; −1.9408, −0.3267, *P* = 0.006). The sum of pain duration in the three days was 18.6 ± 8.8 hours in the first group versus 23.1 ± 8.9 hours in the second group. This difference was significant (95% CI; −7.9121, −0.7519, *P* = 0.018). 

### 3.3. Changes of Pain Level and Duration in the Second Treatment Phase

 In the second treatment phase, both groups switched to alternate regimen. The second phase of the treatment also represented a significantly lower VAS-pain and pain duration in the second group as compared to the first group (Table 4). The VAS-pain in the first day was 5.7 ± 2 in the second group compared to 6.8 ± 1.9 in the first group. This difference was significant (95% CI; 0.2449, 1.8413, *P* = 0.011). In the second day, the VAS-pain was also significantly lower in the second group (4.4 ± 2) than in the first group (5.5 ± 2.1) (95% CI; 0.2789, 1.9294, *P* = 0.009). In the third day, the second group continued to have significantly lower average VAS-pain when compared to the first group (2.6 ± 2 versus 4.1 ± 2.6, resp.) (95% CI; 0.5758, 2.4611, *P* = 0.002). The sum of pain duration in the three days was 24.5 ± 8.89 hours in the first group versus 19.3 ± 9.6 hours in the second group. This difference was significant (95% CI; 1.4651, 8.8957, *P* = 0.007). 

### 3.4. The Outcomes of the Menstrual Bleeding during the Two Phases among the Two Groups

Thirty-three of the 95 students in the current study reported excessive menstrual bleeding at baseline assessment (they were 17 and 16 students in group 1 and group 2, resp.). We evaluated the effect of aromatherapy on excessive menstrual bleeding for those students. The use of aromatherapy in the first treatment phase has turned more students from excessive to average menstrual bleeding in the first day (29.4% in group 1 versus 0% in group 2, *P* = 0.019) and in the second day (64.7% in group 1 versus 12.5% in group 2, *P* = 0.002) (Table 3). 

 Also, during the second treatment phase, the aromatherapy have turned more students in group two from excessive to average menstrual bleeding in the first day (31.3% in group 2 versus 0% in group 1, resp., *P* = 0.012) and in the second day (62.5% in group 2 versus 17.6% in group 1, resp., *P* = 0.008). However, for both treatment phases there was no significant difference for the third day among the two groups (Table 5).

## 4. Discussion

This is one of the first Egyptian nursing research studies using randomized clinical trial of crossover design to investigate the effect of aromatherapy abdominal massage on alleviating primary dysmenorrhea and reducing its duration and excessive menstrual bleeding. Menstrual pain is subjective and personal topics women may suffer from it without seeking help from health care professionals [20–22]. However, for some women, the internal pressure of the uterine contraction during menstruation may be higher than that of labor [23].

The results of this study demonstrates that students used aromatherapy massage in both treatment phases showed greater reduction of pain and its duration during the first three days of menstruation. This result supports previous findings that aromatherapy massage alleviates menstrual pain compared with placebo oil massage [11, 24, 25]. However, our result is not supporting the effect of massage on its own as the 2005 Korean study that involved abdominal massage for 5 minutes per day during 6 days starting five days before menstruation to the first day of menstruation. Their results demonstrated that abdominal massage was a very effective treatment for dysmenorrhea. In the same line, Hur et al., 2011 [26] could not verify whether the positive effects in alleviating menstrual pain were derived from aromatherapy, massage, or both.

Randomized blind placebo clinical trial of aromatherapy massage shows that students who used massage with placebo oil as the same massage applied for aromatherapy students experienced more pain level than those who used massage with aromatherapy. It proves that the positive effects were mainly due to aromatherapy and not to the massage.

Effectiveness of aromatherapy in alleviating the menstrual pain may be attributed to more than one action; generally aromatherapy triggers the limbic system which might help in reducing the pain, also abdominal massage using essential oils may improve the blood circulation as well decreasing the spasm that causes pain. In addition to the effect of lavender as an analgesic, sedative, and anticonvulsant, the effect of clove in increasing the circulation may relieve the pain of smooth muscles as well as its local anesthetic effect, and the effect of cinnamon inhibits the prostanoid system which is involved in the production of PGE2 [27].

Also, the results of this study demonstrates that using aromatherapy turned more students from excessive to average menstrual bleeding in the first and second days but not in the third day compared with students in the placebo group who used almond oil. Our results may be attributed to the effect of cinnamon which helps to treat excessive menstrual bleeding and to support the actions of estrogen, and rose which has a great effect on the uterus and helps regulate the menstrual cycle and reduce excessive bleeding [17, 28].

This study has two limitations: the first one is that the therapeutic effects of essential oils can be different according to the country or region of harvest of the plants because plants grown in different areas can contain oils with different chemotypes or that contain different proportions of chemicals. The second limitation is the scanty of studies that investigated the efficacy of aromatherapy massage on reducing excessive menstrual bleeding.

In conclusion, this research may suggest that aromatherapy has a significant effect on pain and bleeding during menstruation. Because there were no side effects reported, aromatherapy can be regarded as a safe and effective treatment for primary dysmenorrhea. There is a great potential for more collaborative research by nurses to explore the clinical applications in greater details and to move beyond the low dose paradigm of application of essential oils. Also, more researches are recommended to study the efficacy of aromatherapy massage on reducing menstrual bleeding on a large scale studies. 

## Figures and Tables

**Table 1:** Student's baseline data at the time of enrollment.

	Group 1 (*n* = 48)	Group 2 (*n* = 47)	*P*
Age at randomization (year)	20.1 ± 2.2	19.9 ± 1.8	0.542
BMI (kg/m^2^)	23.2 ± 1.8	22.7 ± 1.9	0.133
Age at menarche (years)	12.5 ± 1.7	12.4 ± 1.7	0.778
Length of menstrual cycle (days)	27.5 ± 4.5	28 ± 4.2	0.578
Duration of menstrual flow (days)	4.9 ± 1.5	4.5 ± 1.3	0.259
VAS-pain			
1st day	6.7 ± 1.8	6.8 ± 1.6	0.835
2nd day	5.5 ± 2.2	5.3 ± 1.9	0.682
3rd day	4.1 ± 2.6	3.9 ± 2.4	0.733
Duration of pain (hours)	24.6 ± 8.9	23.1 ± 8.9	0.408
Amount of menstrual blood (number of pads)			
1st day			
Scanty (1 pad)	6 (12.5%)	7 (14.9%)	0.943
Average (2-3 pads)	25 (52.1%)	24 (51.1%)	
Excessive (≥4 pads)	17 (35.4%)	16 (34%)	
2nd day			
Scanty (1 pad)	8 (16.7)	8 (17%)	0.995
Average (2-3 pads)	26 (54.2%)	25 (53.2%)	
Excessive (≥4 pads)	14 (29.2%)	14 (29.8%)	
3rd day			
Scanty (1 pad)	12 (25%)	12 (25.5%)	0.907
Average (2-3 pads)	33 (68.8%)	31 (66%)	
Excessive (≥4 pads)	3 (6.3%)	4 (8.5%)	

**Table 2:** Comparison of the VAS-pain and duration of pain between the two groups at the first treatment phase.

	Group 1 (*n* = 48)	Group 2 (*n* = 47)	95% CI	*P*
VAS-pain				
1st day	5.8 ± 2.1	6.8 ± 1.7	−1.7921, −0.2213	0.013
2nd day	4.3 ± 2	5.4 ± 1.9	−1.7359, −0.1502	0.010
3rd day	2.7 ± 2	3.8 ± 1.9	−1.9408, −0.3267	0.006
Duration of pain (hours)	18.6 ± 8.8	23.1 ± 8.9	−7.9121, −0.7519	0.018

**Table 3:** Comparison of the effect of aromatherapy versus placebo on the amount of menstrual blood in students with excessive menstrual bleeding at the first treatment phase.

	Group 1 (*n* = 17)	Group 2 (*n* = 16)	*P*
1st day			
Turn to average	5 (29.4%)	0	0.019
Remain excessive	12 (70.6%)	16 (100%)	
2nd day			
Turn to average	11 (64.7%)	2 (12.5%)	0.002
Remain excessive	6 (35.5%)	14 (87.5%)	
3rd day			
Turn to average	16 (94.1%)	12 (75%)	0.126
Remain excessive	1 (5.9%)	4 (25%)	

**Table 4:** Comparison of the VAS-pain and duration of pain between the two groups at the second treatment phase.

	Group 1 (*n* = 48)	Group 2 (*n* = 47)	95% CI	*P*
VAS-pain				
1st day	6.8 ± 1.9	5.7 ± 2	0.2449, 1.8413	0.011
2nd day	5.5 ± 2.1	4.4 ± 2	0.2789, 1.9294	0.009
3rd day	4.1 ± 2.6	2.6 ± 2	0.5758, 2.4611	0.002
Duration of pain (hours)	24.5 ± 8.89	19.3 ± 9.6	1.4651, 8.8957	0.007

**Table 5:** Comparison of the effect of aromatherapy versus placebo on the amount of menstrual blood in students with excessive menstrual bleeding at the second treatment phase.

	Group 1 (*n* = 17)	Group 2 (*n* = 16)	*P*
1st day			
Turn to average (2-3 pads)	0	5 (31.3%)	0.012
Remain excessive (≥4 pads)	17 (100%)	11 (68.8%)	
2nd day			
Turn to average (2-3 pads)	3 (17.6%)	10 (62.5%)	0.008
Remain excessive (≥4 pads)	14 (82.4%)	6 (37.5%)	
3rd day			
Turn to average (2-3 pads)	12 (70.6%)	15 (93.8%)	0.085
Remain excessive (≥4 pads)	5 (29.4%)	1 (6.3%)	
